# *COASY*-Associated Disorders as a Differential Diagnosis in Cases with Newborn Screening Results Suggestive of CPT-I

**DOI:** 10.3390/ijns12020025

**Published:** 2026-04-17

**Authors:** Zinandré Stander, Amy L. White, Matthew Lynch, David Coman, Justin Rosati, Diana Bailey, Jessica Johnson, Bo Hoon Lee, ChinTo Fong, Joseph Orsini, Matthew J. Schultz, Devin Oglesbee, Dimitar Gavrilov, Dietrich Matern, Patricia L. Hall, Silvia Tortorelli

**Affiliations:** 1 Biochemical Genetics Laboratory, Department of Laboratory Medicine and Pathology, Mayo Clinic, Rochester, MN 55905, USA; stander.zinandre@mayo.edu (Z.S.);; 2 Queensland Lifespan Metabolic Medicine Service, Queensland Children’s Hospital, Brisbane, QLD 4101, Australia; 3 School of Medicine, University of Queensland, Brisbane, QLD 4072, Australia; 4 Department of Neurology, University of Rochester Medical Center, Rochester, NY 14642, USA; 5 New York State Department of Health, Albany, NY 12208, USA

**Keywords:** *COASY*-related disorders, pontocerebellar hypoplasia type 12, *COASY* protein-associated neurodegeneration with brain accumulation, newborn screening, carnitine palmitoyltransferase I deficiency, co-enzyme A synthase

## Abstract

*COASY*-related disorders (CRDs) are a spectrum of autosomal recessive conditions caused by the dysfunction of CoA synthase, an enzyme responsible for the final steps of CoA synthesis. Clinical manifestations of CRDs are highly variable, ranging from perinatal lethal pontocerebellar hypoplasia to childhood-onset neurodegenerative brain iron accumulation, which is often recognized after clinical regression. Recent reports have described a few individuals with CRD who screened positive for carnitine palmitoyltransferase-I deficiency by newborn screening (NBS). However, heterogeneous clinical presentations, conflicting biochemical/molecular sequencing of CPT1A, and a lack of metabolic characterization have led to lengthy, costly diagnostic journeys. To address some of these aspects, this investigation retrospectively evaluated NBS acylcarnitine patterns in five CRD cases using Collaborative Laboratory Integrated Reports (CLIR). A total of 25 metabolites/ratios were identified to deviate significantly from reference ranges and were primarily composed of elevated free carnitine and reduced long-chain acylcarnitine levels. While low acylcarnitine concentrations are often not reported due to a lack of lower reference cutoffs, ratios involving these metabolites relative to short-chain acylcarnitines could aid in identifying CRD cases via NBS. When comparing this pattern to CPT-Ia cases, we confirmed a nearly identical acylcarnitine pattern between these, and thus support the need to consider CRD in cases with NBS results suggestive of CPT-Ia. This study is the first case series to characterize NBS patterns in patients with CRD and highlights the unique opportunity for early detection, particularly in cases that are neonatally asymptomatic and have unremarkable confirmatory biochemical results.

## 1. Introduction

Coenzyme A (CoA) is a ubiquitous cofactor required in a plethora of anabolic and catabolic reactions in the human body. De novo synthesis of CoA constitutes a highly conserved, five-step process that starts with the phosphorylation of pantothenate (vitamin B5) and accounts for the majority of the CoA content in the body [1,2]. Considering the central role of this cofactor, it is unsurprising that impaired functionality in any enzyme of this pathway leads to several debilitating disorders [3,4,5]. CoA synthase (encoded by *COASY*), a bifunctional enzyme that catalyzes the final two steps of de novo CoA synthesis [3], is the most recently implicated enzyme. It has been described in several autosomal recessive conditions, including pontocerebellar hypoplasia type 12 (PCH12; OMIM: 618266) [5,6,7], *COASY* protein-associated neurodegeneration (CoPAN; OMIM: 615643) [8,9,10], and, more recently, riboflavin-responsive lipid storage myopathy (RR-LSM) [4]. While the clinical manifestations of *COASY*-related disorders (CRD) appear distinct, recent reports suggest that these disorders may instead represent a continuum of clinical phenotypes, ranging from perinatal lethal PCH12 with microcephaly and arthrogryposis, to a slow-progressive, childhood-onset CoPAN presentation, including oromandibular spasticity/dystonia, quadriparesis, and dysarthria [8] that may overshadow less severe RR-LSM-like manifestations [4]. Since the identification of CoPAN as the first CRD, functional studies utilizing model organisms (yeast, zebrafish, drosophila, and mice) and patient fibroblasts have provided further insights into the pathophysiology of these disorders, particularly in relation to mitochondrial respiration, iron hemostasis, oxidative stress, transport, and molecular signaling [8,10,11,12,13]. While altered RNA/CoA synthase protein expression and a reduction in enzyme activity may at least partly contribute to the pathophysiological impacts of CRD [10,13], studies involving both CoPAN patient fibroblasts and neuronal-ablation models have revealed unaffected total CoA content. This suggests that, pending the molecular complement, residual enzyme activity may be sufficient to maintain CoA levels in certain individuals and/or that alternative pathways of CoA biosynthesis and recycling may exist [8,10,11]. As a result, there is currently no recognized unified biochemical signature for CRD detection in a clinical setting. This makes the diagnostic process primarily reliant on molecular genetic analysis of *COASY* in the presence of specific clinical features.

Interestingly, a handful of case reports have uncovered abnormal NBS results suggestive of carnitine palmitoyltransferase I deficiency (CPT-Ia; OMIM: 600528) in CRD cases. NBS findings were primarily significant for elevated free carnitine (C0), and an increased ratio of C0 relative to the sum of palmitoyl- and stearoylcarnitine (C16/C18) in the absence of carnitine supplementation, though confirmatory plasma acylcarnitine testing was usually unremarkable [6,9,14]. Although these abnormal NBS results may initially perplex clinical teams, particularly in cases with early-onset PCH12 manifestations that lead to costly, time-consuming diagnostic workup, it could also provide a unique opportunity for early detection of CRD. This would not only reduce diagnostic delays but also allow for timely genetic counseling of the family. Early detection may be particularly helpful in CoPAN cases that are asymptomatic immediately after birth and only present for diagnostic evaluation after notable clinical regression.

This investigation is the first case series to explore and characterize the NBS acylcarnitine patterns in CRD cases, aiming to better understand the metabolic signatures associated with these conditions and their potential implications for early diagnosis and clinical management.

## 2. Materials and Methods

### 2.1. Study Design and Ethics

**Figure 1:**
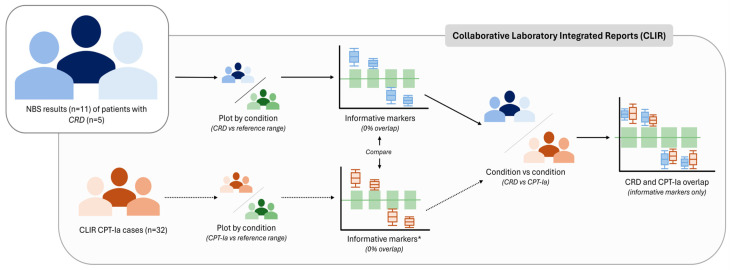
Summary of workflow employed in this investigation. Abbreviations: CLIR—Collaborative Laboratory Integrated Reports, CPT-Ia—Carnitine palmitoyltransferase I deficiency, CRD—*COASY*-related disease, and NBS—Newborn screening. Footnote: * Markers considered ‘informative’ if 0% overlap, though may not all be implemented on a clinical basis for CPT-Ia.

The study design of this investigation is presented in Figure 1, and relevant components are discussed in the sections below. In summary, it involves a retrospective review of CPT-Ia-positive NBS results from five subjects (three unrelated families) with molecularly confirmed CRD. NBS data was obtained from the New York State Department of Health and Queensland Public Health NBS programs, and post-analytical review was conducted using Collaborative Laboratory Integrated Reports (CLIR; https://clir.mayo.edu). Clinical information was provided by collaborating primary care teams at the University of Rochester Medical Center, NY, USA and Queensland Children’s Hospital, South Brisbane, Australia. This study falls under a larger minimal risk ethics application (ID# 21-004405) approved by the Mayo Clinic Institutional Review Board (IRB). Written consent for review of medical records and publication of findings was obtained from legal representatives of all patients prior to commencement of the study.

### 2.2. Patient Clinical History

Pertinent clinical and molecular information of the cases included in this investigation is summarized in Table 1, while their respective clinical courses are briefly described below:

#### 2.2.1. Family 1

Two siblings (Subjects 1.1 and 1.2) previously described by Rosati et al. [6]. In summary, subjects independently presented with hyperglycemia, severe hypotonia, poor Moro and deep tendon reflexes, and respiratory insufficiency immediately after birth. Brain magnetic resonance imaging (MRI) on day of life (DOL) 2 revealed symmetric diffusion restriction in the hippocampi, globus pallida, thalami, and posterior limbs of the internal capsules in both cases. Although NBS results were suggestive of CPT-Ia, targeted molecular analysis of *CPT1A* was unremarkable. Upon further, more comprehensive molecular investigation, i.e., whole exome sequencing (WES), a homozygous variant of uncertain significance (Table 1) in *COASY* was identified in both cases.

#### 2.2.2. Family 2

Two siblings (Subjects 2.1 and 2.2), of which Subject 2.2 was part of a separate dizygotic (dichorionic, diamniotic) twin pair [14]. Subjects 2.1 and 2.2 were born 29 months apart to the same non-consanguineous Caucasian parents. Affected siblings had similar clinical manifestations, which initially included microcephaly, increased limb tone, absent deep tendon reflexes, and refractory epilepsy within the first two DOL. Nasogastric feeding was required in both cases due to poor suckling reflexes. Brain MRIs revealed a hypoplastic cerebellum, immature cortical sulcation, parenchymal atrophy, and a dilated ventricular system in both siblings. While both Subjects had positive NBS suggestive of CPT-Ia, confirmatory plasma evaluations of Subject 2.1 (performed on DOL 12) revealed unremarkable acylcarnitine levels with only mildly elevated C0, while all biochemical testing for Subject 2.2 (performed on DOL 4) was normal. Considering opposing clinical and biochemical findings, WES was performed and revealed compound heterozygous variants in *COASY*. Unfortunately, Subject 2.1 developed necrotizing enterocolitis on DOL 17 and died on DOL 41. Subject 2.2, on the other hand, developed feeding intolerance and recurrent intestinal pseudo-obstructions at the age of 17 months and died shortly thereafter.

#### 2.2.3. Family 3

Female singleton recently described by Lynch et al. [14]. Subject 3.1 was born to non-consanguineous Caucasian parents following induction of labor due to maternal gestational diabetes. Besides requiring phototherapy for unconjugated hyperbilirubinemia, the infant appeared healthy with no immediate other clinical concerns until an abnormal NBS result suggestive of CPT-Ia was reported. Confirmatory plasma acylcarnitine analysis was unremarkable. Clinical regression became apparent soon thereafter with mild motor developmental delays, followed by focal seizures at 15 months of age. A subsequent brain MRI demonstrated a thin corpus callosum, with symmetric T2-weighted imaging hyperintensity and diffusion restriction in the basal ganglia and thalami. At 19 months of age, dystonia was present in the lower limbs, with evidence of brisk deep tendon reflexes in both upper and lower limbs. Hereafter, comprehensive molecular testing (WES) was performed and revealed compound heterozygous variants in *COASY* (Table 1).

### 2.3. NBS Data Analysis

Flow-injection tandem mass spectrometry (FIA-MS/MS; derivatized or underivatized) acylcarnitine results of all patients were analyzed using Collaborative Laboratory Integrated Reports (CLIR; https://clir.mayo.edu). CLIR is a web-based multivariate pattern recognition tool that assists in post-analytical result interpretation by comparing patient results to covariate-adjusted disease and reference ranges [16,17,18]. CLIR was employed to collectively compare CRD NBS data with well-established reference ranges that consist of NBS results from >10,000 reference samples from multiple laboratories. Metabolites were deemed “informative” if deviating significantly (0% range overlap) from these reference ranges and constitute/include markers that are more commonly measured by NBS programs to ensure unbiased utility. Given the small cohort available, repeat NBS measures were included in this investigation. CDR (*n* = 11) and CPT-Ia cases (*n* = 32) were evaluated using several available productivity tools in CLIR, primarily the Plot by Condition and Plot by Multiple Conditions functionalities, which allow comparison of single/multiple conditions with the cumulative reference ranges for key analytes.

## 3. Results

**Figure 2:**
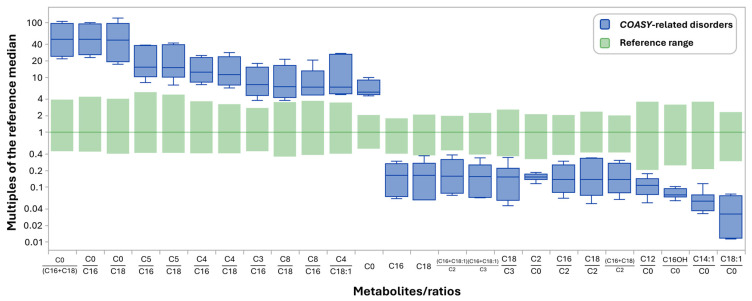
Collaborative Laboratory Integrated Reports (CLIR) metabolites/ratios that deviate significantly (0% overlap) between COASY-related disorders (CRD) and reference ranges. Abbreviations: C0—Free carnitine, C2—Acetylcarnitine, C3—Propionylcarnitine, C4—Butyryl-/Isobutyrylcarnitine, C5—Valeryl-/isovalerylcarnitine, C8—Octanoylcarnitine, C12—Dodecanoylcarnitine, C14:1—Tetradecenoylcarnitine, C16—Palmitoylcarnitine, C16OH—Hydroxypalmitoylcarnitine, C18—Stearoylcarnitine, and C18:1—Oleoylcarnitine.

Retrospective review of the NBS profiles (*n* = 11) for the five cases included in this investigation revealed at least 39 acylcarnitine species/ratios that deviate significantly (0% overlap) from the established reference ranges (Figure S1). Of these, 25 are more commonly measured by NBS programs (Figure 2), while the remaining include hydroxyacyl and dicarboxylic acylcarnitine species (Figure S1) that are not consistently measured and/or subdivided according to derivatization methods [19]. As for informative markers, the majority of these appear to be related to an increase in C0 and a concomitant reduction in C16 and C18 acylcarnitines. Hence, ratios including C0/(C16+C18), C0/C16, and C0/C18 are most useful and show the greatest deviation from the reference range, similar to what is seen in CPT-1a and in false-positive cases due to carnitine supplementation. Since not all NBS programs employ low reference cutoffs or report reductions in acylcarnitine species, such as C16 and C18, ratios to other abundant acylcarnitine species, including short-chain (C3–C5) and medium-chain (C8) acylcarnitines, may prove useful for identifying cases with CRD.

**Figure 3:**
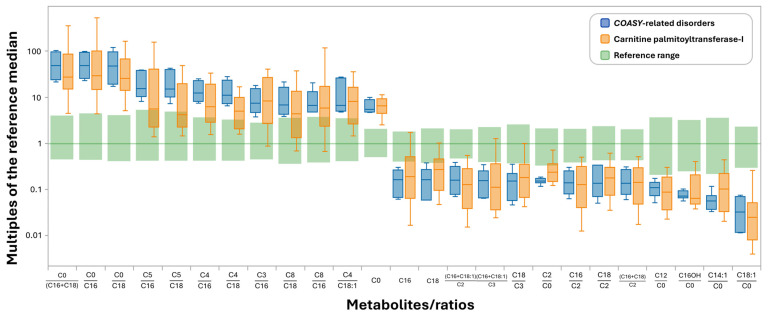
Comparison of metabolite pattern overlap between CPT-Ia and CRD cases using Collaborative Laboratory Integrated Reports (CLIR). Metabolites/ratios shown are those that deviate significantly (0% overlap) between CRD and reference ranges. Footnote: Due to the limited size of the CRD-cohort, these plots are not covariate-adjusted. Abbreviations: C0—Free carnitine, C2—Acetylcarnitine, C3—Propionylcarnitine, C4—Butyryl-/Isobutyrylcarnitine, C5—Valeryl-/isovalerylcarnitine, C8—Octanoylcarnitine, C12—Dodecanoylcarnitine, C14:1—Tetradecenoylcarnitine, C16—Palmitoylcarnitine, C16OH—Hydroxypalmitoylcarnitine, C18—Stearoylcarnitine, C18:1—Oleoylcarnitine,.

When comparing NBS results for CRD and CPT Ia patients collectively (Figure S2), it is clear that there is notable overlap in markers that differentiate these diseases from the reference ranges. While several metabolites/ratios included in the CRD informative pattern (Figure S1) were not observed in the CPT-1a pattern (Figure S2) and vice versa, it was established that several of these were excluded from the marker list due to the 0% overlap cut-off that was applied (Figure 3 and Figure S3). Finally, to evaluate the degree of overlap between the diseases for all relevant informative markers, disease ranges were collectively compared and presented in Figure 3. From this, it is clear that the metabolic pattern of CRD is exceedingly similar to that of CPT-Ia and highlights the potential of identifying CRD, which should be considered in the workup of neonates with NBS results suggestive of CPT-Ia and absence of obvious confounders such as carnitine supplementation.

## 4. Discussion

Here, we present a cohort of five newborns with molecularly confirmed CRD (Table 1), originally identified due to abnormal NBS results suggestive of CPT-Ia and complex perinatal clinical manifestations [6,14]. Using NBS data from these cases, this retrospective investigation aimed to provide the first characterization of the NBS pattern in CRD cases and to assess the degree of overlap with positive CPT-Ia NBS patterns.

Comparing the NBS results (*n* = 11; including repeat testing) of the CRD cases to well-established reference ranges using CLIR [16,17,18], approximately 25 metabolites/ratios informative markers deviated significantly (Figure 2). These markers are mostly related to a concomitant C0 elevation and long-chain acylcarnitine (C16 and C18) reduction, with C0/C16+C18, C0/C18, and C0/C16 being the most prominent. Since *COASY* encodes the bifunctional enzyme responsible for the final two steps of CoA synthesis, impaired functionality of this enzyme may reduce the CoA availability [10,13] required for numerous processes, including transport of long-chain fatty acids into the mitochondria and subsequent ꞵ-oxidation [20]. In turn, dysregulation of long-chain acyl-CoA production may affect carnitine shuttling, potentially leading to an increase in free carnitine availability, as observed in these cases. While the elevation of C0 and related ratios may serve a useful purpose, a reduction in respective long-chain acylcarnitines may not be universally applicable, as not all NBS programs have lower reference range cut-offs for reporting reductions in acylcarnitine species. However, incorporating these reductions in ratios relative to other abundant acylcarnitine species may prove more applicable. Based on the NBS patterns observed in this investigation, several such ratios are evident (Figure 2) and mainly include those related to short- and medium-chain acylcarnitines. Short- and medium-chain fatty acids are likely less impacted by this disease due to their hydrophilic nature, which enables them to cross mitochondrial membranes independently of the carnitine shuttling system [20], making these less dependent on CoA than long-chain fatty acids for transport [21]. It is plausible that CoA conjugation of these fatty acids within the mitochondria prior to oxidation [21] may be accounted for by the residual CoA synthase activity observed in these cases, as complete loss of function is likely incompatible with life [3,11].

Interestingly, this overall pattern is not entirely unfamiliar to NBS laboratories (Figure S2), as concomitant elevations of C0 and C0/C16+C18 are considered a characteristic finding in cases with CPT-Ia [22,23] and often seen in false positive cases due to carnitine supplementation and, more recently, in a small group of Pantothenate Kinase-Associated Neurodegeneration (PKAN) cases [24]. CPT-Ia is an autosomal recessive mitochondrial fatty acid β-oxidation disorder included as a time-critical core condition on the Recommended Uniform Screening Panel (RUSP; https://www.hrsa.gov/advisory-committees/heritable-disorders/rusp; accessed 7 March 2025) in the USA that results in impaired long-chain fatty acid transport into the mitochondria for β-oxidation, leading to hypoketotic hypoglycemia, lethargy, hepatomegaly, and seizures triggered by fasting and/or acute illness due to dysfunction of CPT and subsequent acylcarnitine formation [20,22]. Although this differs from the pathological mechanisms associated with CRD, the patterns likely appear similar due to the types of analytes measured. Figure 3 provides a visual representation of the degree of profile overlap of these disorders and further substantiates why these patient results were reported as suggestive of CPT-Ia by state NBS programs. However, it is important to recognize that false-positive elevations in C0 and related metabolites due to carnitine supplementation remain common confounding factors in the interpretation of acylcarnitine profiles.

While CRD do not meet the minimum requirements to be included in the RUSP as a primary screening target condition [25], the potential overlap with the CPT-Ia could provide a unique window of opportunity for early identification of individuals with CRD, as plasma/fibroblast C0 and acylcarnitine levels in these cases tend to be unremarkable [8,9,10,14]. This is evident not only in the literature but also in samples from cases included in this investigation (Table 1) and has been ascribed to potential alternative compensatory mechanisms that are not yet fully understood [8,11]. Hence, depending on the severity of clinical manifestations (i.e., PCH12 vs. CoPAN), diagnostic evaluations may only be initiated upon significant clinical regression, leading to an overall delay in a patient’s diagnosis, enrollment in potential future clinical trials, and appropriate genetic counseling for the family.

Conversely, the overlap of these biochemical patterns and conflicting clinical presentations (particularly in cases with PCH12) often leads to laborious clinical workups that are invasive, costly, and time-consuming [6,9,14]. Furthermore, since current guidelines for a positive NBS suggestive of CPT-Ia recommend follow-up biochemical confirmatory testing and/or targeted *CPT1A* sequencing [23], asymptomatic individuals with normal plasma/fibroblast testing or unremarkable sequencing testing may be dismissed as false-positive NBS results. Unfortunately, although a small number of markers/ratios have been identified as potentially distinguishing these disorders from one another [26], some of these markers are yet to be routinely measured by most NBS programs and are based on limited sample sizes, which present challenges for their clinical implementation at this time. Hence, until these can be differentiated, it is important that laboratories and clinical teams understand the potential implications of a positive NBS result suggestive of CPT-Ia and consider CRD as a possible secondary diagnosis in infants for which a false-positive NBS result due to carnitine supplementation has been ruled out.

The authors recognize several limitations of this investigation: (1) Considering the rare nature of CRD, only a small cohort of patients with restricted clinical phenotypes (the majority being PCH12) and repeated NBS is included in this study. Hence, future investigation consisting of larger cohorts and a broader spectrum of clinical phenotypes may be useful in supporting the patterns observed in these patients; (2) Since NBS for these patients was performed by different screening laboratories, markers not concomitantly measured by institutes, and those dependent on derivatization methods were disregarded.

## 5. Conclusions

This investigation presents the first case series to explore and characterize the NBS metabolic acylcarnitine patterns of cases with CRD. From these results, it is clear that NBS may provide a unique opportunity for identifying these patients as a collective secondary target condition, and emphasizes the need for considering and evaluating *COASY*-related genetic variants in cases with NBS results suggestive of CPT-Ia (absence of carnitine supplementation) and clinical findings suggestive of this spectrum of disorders.

## Figures and Tables

**Table 1:** Clinical characteristics of the patients included in this investigation.

Cases		Sex	Gestational Age	Birthweight	Clinical Diagnosis	Follow-Up Biochemical Testing	Molecular Variants in *COASY*
			(Weeks)	(Grams)			
**Family 1**	Subject 1.1	Female	39	3047	PCH12	**Plasma:** Elevated C0 Unremarkable acylcarnitines	**Homozygous:** NM_025233.6:c.1664G>A (p.Arg555His) **Classification:** Variant of uncertain significance
	Subject 1.2	Female	36	2337			
**Family 2**	Subject 2.1	Female	37	2200	PCH12	**Plasma:** Normal/mildly elevated C0 Unremarkable acylcarnitines	**Compound heterozygous:** NM_025233.6: c.215A>G (p.Tyr72Cys) **Classification:** Variant of uncertain significance NM_025233.6: c.1403_1404dup (p. Ile469*) **Classification:** Pathogenic
	Subject 2.2	Male	36	2530			
**Family 3**	Subject 3.1	Female	41	2680	CoPAN	**Plasma:** Mildly elevated C0 Unremarkable acylcarnitines	**Compound heterozygous:** NM_025233.6: c.1403_1404dup (p. Ile469*) **Classification:** Pathogenic NM_025233.6: c.1495C>T (p.Arg499Cys) **Classification:** Pathogenic

Abbreviations: c.—Coding DNA, C0—Free carnitine, CoPAN—COASY protein-associated neurodegeneration, NM—Molecular transcript, PCH12—Pontocerebellar hypoplasia type 12, p.—Protein level and *—Termination (Stop) codon. Footnote: Molecular classification updated based on ACMG classification guidelines [15] for standardization purposes.

## Data Availability

Data generated during this investigation have been included in this published article and/or the Supplementary Materials. Additional data requests can be directed to the corresponding authors.

## Supplementary Materials

The following supporting information can be downloaded at: https://www.mdpi.com/article/10.3390/ijns12020025/s1, Figures S1–S3. Figure S1—Comprehensive list of metabolites/ratios that deviate significantly (0% overlap) between CRD and reference ranges. Figure S2—Metabolites/ratios that deviate significantly (0% overlap) between CPT-Ia cases (*n* = 32) and reference ranges. Figure S3—Metabolites/ratios identified in CPT-1a profiles that have >0% overlap with reference ranges in CRD cases.
