# Supplementary material for: COASY-Associated Disorders as a Differential Diagnosis in Cases with Newborn Screening Results Suggestive of CPT-I
Source: Int J Neonatal Screen. 2026 Apr 17;12(2):25. doi: 10.3390/ijns12020025 (PMC13108042; doi:10.3390/ijns12020025)
Supplement: Supplementary file 1 [file IJNS-12-00025-s001.zip › IJNS-4142032-supplementary.pdf]

*Supplementary Materials for:*

# *COASY*-associated disorders as a differential diagnosis in cases with newborn screening results suggestive of CPT-I

Zinandré Stander[1], Amy L. White[1], Matthew Lynch[2], David Coman[3], Justin Rosati[4], Diana Bailey[4], Jessica Johnson[4], Bo Hoon Lee[4], ChinTo Fong[4], Joseph Orsini[5], Matthew J. Schultz[1], Devin Oglesbee[1], Dimitar Gavrilov[1], Dietrich Matern[1], Patricia L. Hall[1], and Silvia Tortorelli[1]

*[1]Biochemical Genetics Laboratory, Department of Laboratory Medicine and Pathology, Mayo Clinic, Rochester, MN, USA; [2]Queensland Lifespan Metabolic Medicine Service, Queensland Children's Hospital, South Brisbane, Queensland, Australia; [3]School of Medicine, University of Queensland, Brisbane, Queensland, Australia; [4]Department of Neurology, University of Rochester Medical Center, Rochester, NY, USA; and [5]New York State Department of Health, Albany, NY, USA.*

**Corresponding author:** Silvia Tortorelli, MD, PhD – tortorelli.silvia@mayo.edu

**Index:**

I. ***Figure S1 –*** *Comprehensive list of metabolites/ratios that deviate significantly (0% overlap) between CR and reference ranges.*

II. ***Figure S2 –*** *Metabolites/ratios that deviate significantly (0% overlap) between CPT-Ia cases (n=32) and reference ranges.*

III. ***Figure S3 –*** *Metabolites/ratios identified in CPT-1a profiles that have >0% overlap with reference ranges in CRD cases.*

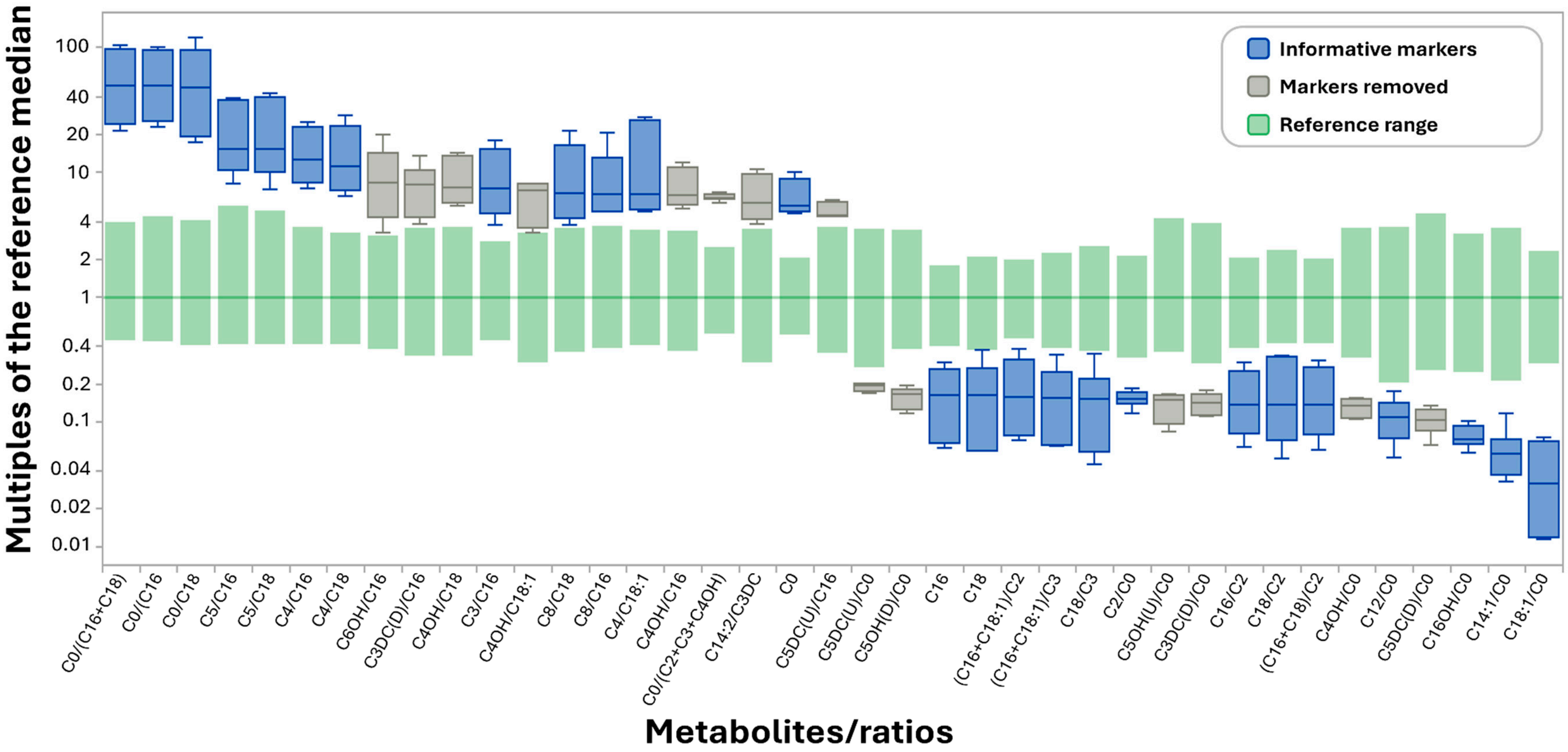


***Figure S1:*** *Comprehensive list of metabolites/ratios that deviate significantly (0% overlap) between COASY-related disorders and reference ranges.* ***Abbreviations:*** *C0 – Free carnitine, C2 – Acetylcarntine, C3 – Propionylcarnitine, C3DC – Malonylcarnitine, C4 – Butyryl/Isobutyrylcarnitine, C4OH – Hydroxybutyrylcarnitine, C5 – Valeryl/isovalerylcarnitine, C5DC – Glutarylcarnitine, C5OH – Hydroxyisovalerylcarnitine, C8 – Octanoylcarnitine, C12 – Dodecanoylcarnitine, C14:1 – Tetradecenoylcarnitine, C16 – Palmitoylcarnitine, C16OH – Hydroxypalmitoylcarnitine, C18 – Stearoylcarnitine, C18:1 – Oleylcarnitine, (D) – Derivatized, and (U) – Underivatized*

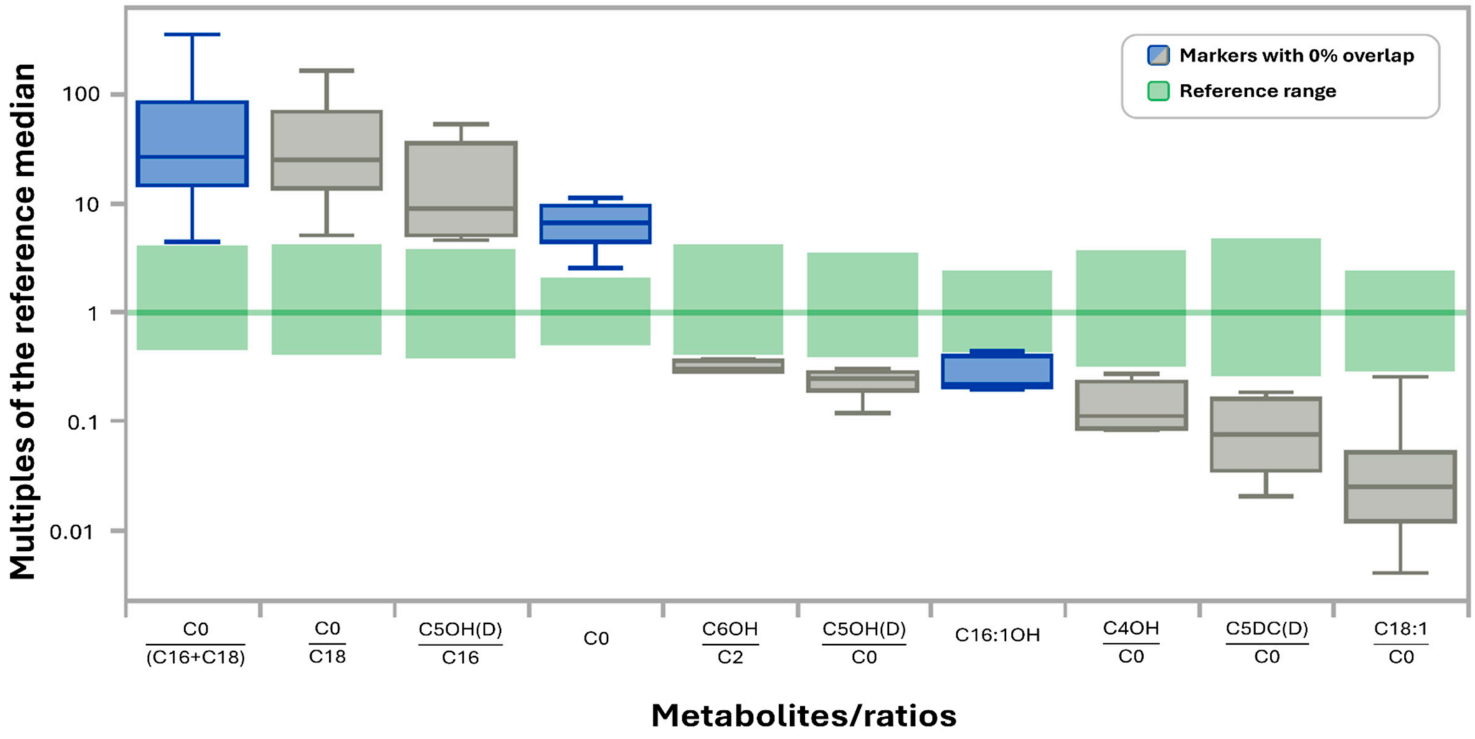


***Figure S2:*** *Metabolites/ratios that deviate significantly (0% overlap) between CPT-Ia cases (n=32) and reference ranges.* ***Abbreviations:*** *C0 – Free carnitine, C2 – Acetylcarntine, C4OH – Hydroxybutyrylcarnitine, C5DC – Glutarylcarnitine, C5OH – Hydroxyisovalerylcarnitine, C16 – Palmitoylcarnitine, C16:1OH – Hydroxypalmitoleylcarnitine, C18 – Stearoylcarnitine, C18:1 – Oleylcarnitine, and (D) – Derivatized.* ***Footnote:*** *Not all 'informative' CPT-Ia markers are currently implemented on a clinical basis, as some of these are different configurations of the same intermediates and/or include markers not commonly measured (grey shaded boxplots)*

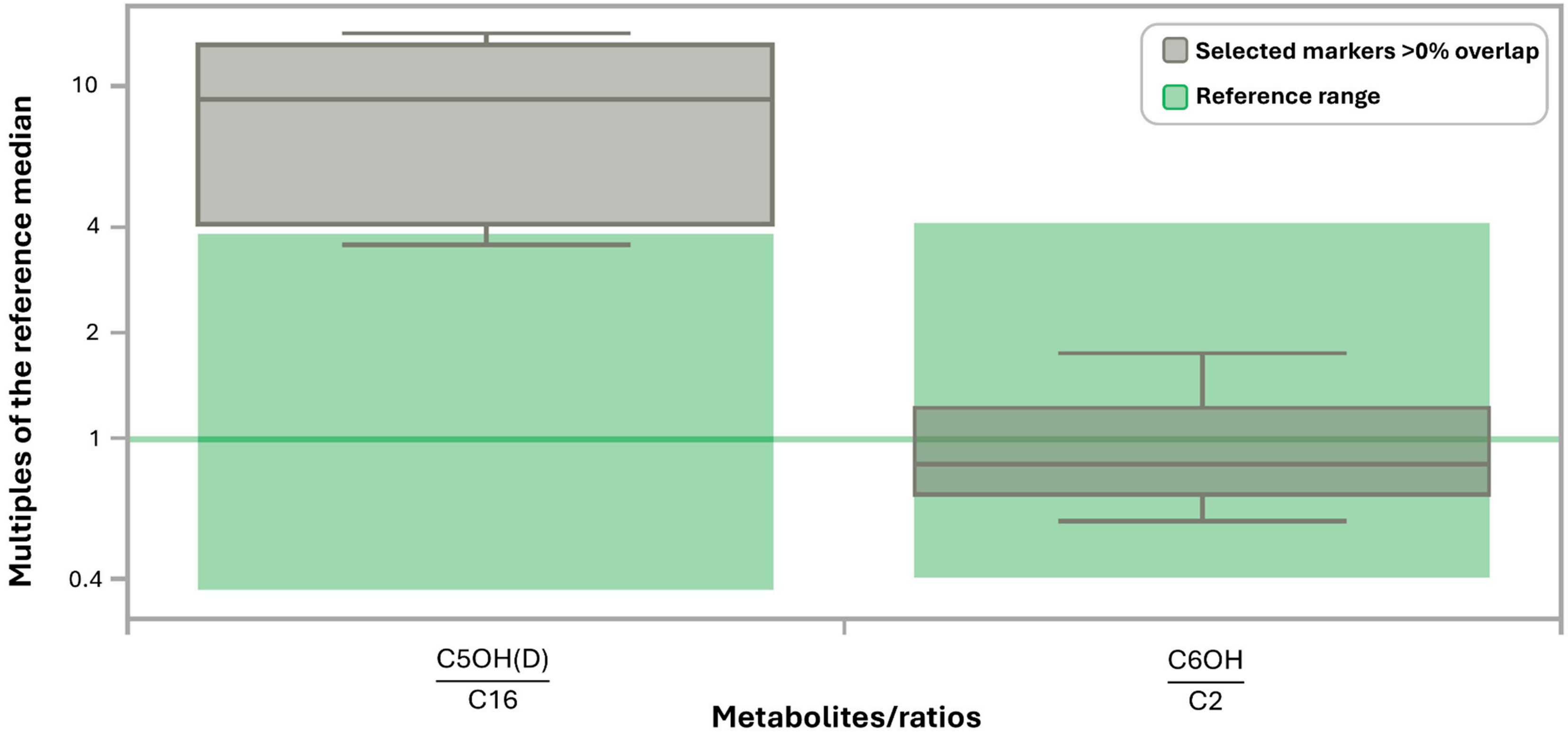


***Figure S3:*** *Markers identified in CPT-1a profiles that have >0% overlap with reference ranges in CRD cases.* ***Abbreviations:*** *C2 – Acetylcarntine, C5OH – Hydroxyisovalerylcarnitine, C6OH – Hydroxyhexanoylcarnitine, C16 – Palmitoylcarnitine, and (D) – Derivatized.* ***Footnote:*** *Since markers have >0% overlap with reference range and/or encompass markers not commonly measured by NBS programs, these are not considered informative (grey shaded boxplots) for CRD identification*
